# Pecans and Human Health: Distinctive Benefits of an American Nut

**DOI:** 10.3390/nu17233686

**Published:** 2025-11-25

**Authors:** Amandeep K. Sandhu, Indika Edirisinghe, Britt Burton-Freeman

**Affiliations:** Center for Nutrition Research, Department of Food Science and Nutrition, Illinois Institute of Technology, Chicago, IL 60616, USA; asandhu2@illinoistech.edu (A.K.S.); iedirisi@illinoistech.edu (I.E.)

**Keywords:** pecans, (poly)phenols, diet quality, cardiometabolic, diabetes, obesity, satiety, brain, gut, microbiome

## Abstract

Pecans are a tree nut native to America with a rich content of unsaturated fatty acids, minerals, fiber, and a diverse array of bioactive components, including polyphenols, tocopherols, and phytosterols. This review summarizes variations in the phenolic composition of pecans from various parts of the world based on cultivar, maturity stage, postharvest storage, and processing. Additionally, the review delves into the bio-accessibility and bioavailability of bioactive components from pecans and their potential influence on diet quality, body weight, satiety, cardiometabolic, brain and gut health. Data from human clinical trials suggest that replacing foods/snacks with pecans improves overall diet quality and lipid profiles. However, inconsistent effects are observed on vascular function, glycemia, and inflammation. Body weight changes after pecan intake are reported as neutral, with promising results on satiety peptides and appetite regulation. Cognition and gut health are emerging areas of research with very limited data from both human and preclinical models, warranting further investigation. Overall, the current literature supports the cardiometabolic benefits of pecans within healthy dietary patterns. Future research should focus on well-controlled studies targeting at-risk populations to understand mechanistic endpoints such as metabolomics, microbiome, and vascular function assessments to substantiate the role of pecans in dietary guidance.

## 1. Introduction

Tree nuts are nutrient-dense/health-promoting foods rich in unsaturated fats, fiber, plant protein, vitamins, minerals, and phytochemicals. Nuts such as almonds, walnuts, pistachios, pecans, and hazelnuts support cardiovascular health and help reduce chronic disease risk [1,2]. Their nutrient and health benefits have been elucidated by evidence-based diets like the Mediterranean and DASH diets.

Pecans (*Carya illinoinensis*) are not only packed with beneficial nutritional qualities but also boast a rich, buttery flavor profile that enhances a wide range of culinary applications. Native to North America, particularly in the Mississippi River Valley and regions of present-day Texas and Mexico, pecans are one of the few major tree nuts indigenous to the United States of America (USA). They were a staple food among many Indigenous peoples for centuries prior to European colonization, prized for their portability, caloric density, and long shelf life. Unlike most other commercially available tree nuts, pecans are intimately tied to the agricultural and cultural heritage of the southern USA, where they have historically played important dietary and economic roles.

Pecans are best known for their iconic presence in desserts such as pecan pie and pralines. However, their use extends into savory applications, snack formulations, and even plant-based dairy alternatives, as chefs and consumers alike explore their rich flavor and nutritional potential. From a health perspective, pecans share many of the cardioprotective benefits associated with other nuts. The U.S. Food and Drug Administration (FDA) issued a qualified health claim stating that consuming 1.5 ounces of most nuts per day, including pecans, may reduce the risk of heart disease [2]. However, pecans have a unique nutritional profile, rich in polyphenols, tocopherols, and plant sterols, which may offer added or synergistic health benefits beyond their fat content.

Although less studied than almonds or walnuts, emerging evidence suggests that pecan consumption offers significant health benefits. This review summarizes current research on pecans and human health, outlining future directions to advance understanding of their role in promoting health. The narrative review begins with an overview of the nutritional and phytochemical composition of pecans, including what is known about the bioavailability and metabolic fate of their major bioactive compounds. This is followed by a detailed review of human clinical studies conducted from 2000 to 2025, focusing on the physiological effects of pecan consumption across a range of cardiometabolic and other health-related outcomes. The authors independently conducted literature searches and organized findings for sharing, cross-referencing, and group discussion.

Relevant studies were identified primarily through literature searches in Medline via PubMed using keywords such as “pecan”, “pecans”, “pecan polyphenols”, “pecans nutrients”, “pecan polyphenols and bioavailability/bio-accessibility”, “pecans polyphenols and storage”, “pecans polyphenols and processing”, “pecans and diet quality”, “pecans and energy/energy intake”, and the combination of “pecans and” with a biological term. These terms included, body weight, overweight, obesity, satiety, cholecystokinin, insulin, glucose, diabetes, metabolic, cardiometabolic, lipids, cholesterol, triglycerides, high density lipoprotein (HDL), low density lipoprotein (LDL), vascular, flow-mediated dilation (FMD), blood flow, endothelial function, heart, blood pressure, brain, cognition, Alzheimer’s disease, neurocognition, neuroscience, longevity, gut, microbiome, and related terms. Additional searches were conducted using Web of Science and Google Scholar, and relevant references were identified through cross-referencing the bibliographies of published papers. A total of 52 articles were selected and included for this review.

## 2. Pecan Production, Cultivars, and Harvesting

Mexico accounts for 44% of the global pecan production, surpassing the USA at 40% (kernel basis, metric tons, 2022/2023) [3]. The other pecan producing countries include South Africa, Australia, Brazil, Israel, and Peru [4]. New Mexico led the USA pecan production, followed by Georgia and Arizona in 2023 [5]. Two types of pecans are produced in the USA which include native and seedling, and improved varieties developed by breeding. There are over 500 pecan cultivars with Desirable, Pawnee, Stuart, Cape Fear, Schley, Moreland, Sumner, Excel, Mahan, Osage, Farley, and Elliot, to name a few cultivars, grown in the USA [6,7]. Some pecan varieties such as Cheyenne, Sioux, Apache, Osage, Pawnee, Mohawk, Kiowa, and Choctaw are named after Native Americans [8]. Pecan harvesting varies depending on the variety, climate, and region. In the USA, the harvesting season begins in late September and extends to December in southeastern and southern regions. Pecans are harvested once the outer husk dries and splits open, allowing the mature nuts to fall to the ground—a visual cue for harvest readiness. Pecans are harvested using commercial harvesters with mechanical arms to shake the nuts off the tree. Harvested pecans are dried to a moisture content of 4% before packaging or storage [9].

## 3. Pecan Nutritive Attributes

Pecans are calorie- and nutrient-dense tree nuts due to their high lipid content, with oleic acid being the most abundant fatty acid, followed by linoleic acid [10,11,12,13,14,15,16,17,18,19,20,21,22]. The proximate composition of pecans varies based on cultivars and geographical location [14,15,21]. For example, among 11 pecan cultivars from southern Brazil, substantial compositional variation was observed, with protein ranging from ~7 to 9%, total dietary fiber from 5.5 to 16%, carbohydrates from ~5 to 17%, lipids from 53 to 70%, and ash from 1.1 to 1.7% [15]. Others have also reported wide variations in macronutrient content when analyzing 10 to 16 different cultivars in different global regions [14,21]. The dominant minerals in pecans include potassium, phosphorus, magnesium, and calcium [23,24,25]. Iron, zinc, copper, manganese, boron, and heavy metals have also been reported in pecan kernels [18]. Sucrose was the predominant sugar, followed by fructose and glucose, in three pecan cultivars from Tunisia [26]. The findings on pecan nutrients from peer reviewed publications align with the USDA food data central (Table 1).

## 4. Pecan (Poly)phenols

Pecans are known not only for their rich content of unsaturated fats but also for a diverse profile of phytochemical components. Among these are (poly)phenols such as ellagitannins, flavan-3-ols (e.g., catechins and epicatechins), and proanthocyanidins [27,28]. Other phytochemicals include carotenoids, tocopherols, and phytosterols [29]. In this section, we discuss the (poly)phenolic content of pecans based on the type of analysis (Supplementary Table S1).

### 4.1. Spectrophotometric and Antioxidant Assays

The (poly)phenol content and antioxidant capacity of pecans have been extensively studied across cultivars and growing regions using a range of spectrophotometric assays. These typically include measurements of total phenolics, anthocyanins, flavonoids, proanthocyanidins, carotenoids, flavanols, and ortho-diphenols. Antioxidant activity is commonly assessed by assays such as ORAC (Oxygen radical absorbance capacity), DPPH (1,1-diphenyl-2-picryl hydrazyl), FRAP (Ferric Reducing Antioxidant Power Assay) and ABTS (2,2′-azinobis-(3-ethylbenzothiazoline-6-sulfonic acid)), and measuring reducing power against hydroxyl radicals.

The phytochemical composition of pecans is influenced by multiple factors such as cultivar, developmental stage, harvest year, soil conditions, agronomic practices, postharvest storage, and processing. Numerous studies have reported significant cultivar-dependent differences in both (poly)phenol content and antioxidant capacity. For instance, “Wichita” often exhibits the highest phenolic content among cultivars [16,30,31]. Phenolic content can decline over time during storage, though antioxidant capacity may persist or increase due to polymerization processes [32,33]. Color darkening, carotenoid loss, and moisture reduction are additional quality changes with storage, while low temperature, controlled moisture, and protective packaging consistently emerge as key strategies for preserving nut quality [34,35].

Maturity stages and growing conditions can also alter the phenolic content. In Tunisian cultivars, total phenolics peaked early and declined with ripening, with Burkett and Mahan cultivars showing the highest total phenolic content at early and mature stages, respectively [36]. Similarly, the Mahan cultivar showed the highest total phenolics, flavonoids, flavanols, and condensed tannins, along with the highest antioxidant activity [36]. Jia et al. (2023) also reported that the phenolic content and antioxidant capacity in Mahan pecans were highest at harvest and declined during storage [32].

Environmental and management practices further influence phenolic composition. Combined mineral and organic fertilization increased total phenolics and antioxidant capacity [37], while mechanical pruning and fruit position within the canopy enhanced antioxidant properties [38].

Pecans generally rank highest in phenolic content and antioxidant capacity when compared to other nuts. They were found to have the highest total phenolics among 10 nut types in Poland [23] and the highest total flavonoids among nuts in a Korean market survey [39]. Another study comparing 11 different kinds of fresh nuts reported walnuts and pecans to be highest in ellagic acid content, total phenol content, and antioxidant capacity [40]. Strong correlations between phenolic concentration and antioxidant capacity have been consistently reported across studies [16,27,36,41].

### 4.2. (Poly)phenol Profiling and Quantification

Advanced analytical techniques, including UHPLC–MS and NMR spectroscopy, have enabled the comprehensive profiling of pecan phytochemicals, which vary from simple phenolic acids to complex tannins [42]. Distribution varies by plant organ, with kernels rich in hydrolysable and condensed tannins, and flavonols more abundant in branches, bark, and leaves [43]. Jia et al. (2023) [32] reported 118 compounds in the Mahan cultivar, including 9 compounds identified for the first time in pecans. Catechin, procyanidin trimers and tetramers, and galloylated glucose derivatives were suggested as quality markers during storage [32]. Epi(catechin) dimers and trimers were identified as the primary bioactive compounds in crude pecan extracts and these compounds were non-cytotoxic in cell-based antioxidant assays, supporting their relevance for human health benefits [44]. In eighteen USA pecan cultivars, gallic acid, ellagic acid, and proanthocyanidins were predominant, with no new aglycones released after hydrolysis [28,31]. Targeted fractionation of pecan extracts has further characterized ellagitannins such as tellimagrandin and pedunculagin [45]. Similar findings on polyphenol profiles were reported in pecans grown in other regions of the world [26,30,35]. Additional metabolites such as protocatechuic acid, p-hydroxybenzoic acid, ellagic acid derivatives, and valoneic acid dilactone have also been identified in pecans [41].

## 5. Bio-Accessibility and Bioavailability

Pecans are rich in various bioactive phytochemicals, but research on their bio-accessibility (the fraction of compounds released during digestion) and bioavailability (the fraction absorbed and available for systemic circulation) is limited. To date, only three studies have examined the bio-accessibility and bioavailability of pecan phytochemicals (Table 2). In vitro digestion assays highlight that the bio-accessibility of pecan phenolics is strongly influenced by molecular size and nut matrix composition. Following gastrointestinal digestion of raw and roasted pecans, low-molecular-weight fractions rich in flavan-3-ols and ellagic acid derivatives decreased substantially, while high-molecular-weight fractions containing procyanidins underwent depolymerization, leading to reduced oligomers and increased dimers. This shift suggests an improved intestinal absorption potential for procyanidin dimers, although overall antioxidant capacity declined in parallel with phenolic losses [46]. The study utilized an in vitro digestion model with isolated, defatted phenolic extracts, which cannot fully replicate the effects of the complex food matrix or the critical role of colonic bacterial metabolism on phenolic bio-accessibility and absorption in the human body.

The plasma concentrations of phenolic components after ingestion of pecans have been reported in two studies. In a crossover clinical trial, 16 healthy men and women (23–44 y, BMI 22.7 ± 3.4 kg/m^2^) consumed meals containing either whole pecans (90 g, ~3 servings), blended pecans (90 g, ~3 servings), or a control meal with a similar macronutrient content without pecans. Various biomarkers in plasma were measured at baseline and at intervals up to 24 h post-ingestion. Plasma concentrations of γ-tocopherols doubled at 8 h (*p* < 0.001) and plasma antioxidant capacity increased at 2 h after the pecan test meals. After whole pecan consumption, plasma epigallocatechin-3-gallate concentrations increased significantly at 1 h (95.1 ± 30.6 nmol/L) and 2 h (116.3 ± 80.5 nmol/L) compared to baseline and the control meal (*p* < 0.05) The limitation of this study was that not all the circulating metabolites were characterized, and those reported were obtained after hydrolysis of the plasma, which does not represent the actual compounds in systemic circulation. Moreover, the assessments were secondary objectives of the study and limited in scope [47]. A recent study reported an increase in urolithin metabolites in human plasma after 4 weeks of pecan consumption. However, the authors did not report any other pecan (poly)phenolic metabolites [48].

Pecans are rich sources of (poly)phenols and other bioactive components; however, there is limited research on how these components are absorbed and metabolized in the human body. Clinical studies linking pecan-derived metabolites to health outcomes are needed to guide optimal intake levels and substantiate their role in dietary guidance for health.

## 6. Diet Quality

The Dietary Guidelines for Americans frame healthy eating in terms of consuming nutrient-dense foods and beverages, which is a central component of diet quality. Nutrient-dense options are those that provide vitamins, minerals, and other beneficial substances with relatively fewer “less desirable” components (added sugars, saturated fat, sodium) [49]. Assessments of the nutrient and phytochemical compositions of pecans reveal a food with health-promoting properties; likewise, including pecans in the diet has been shown to improve diet quality, as measured by the Healthy Eating Index [50]. Hart et al. (2025) and Dos Santos et al. (2022) both reported improved diet quality based on HEI-2020 criteria [51,52]. The HEI-2020 increased by 9.4 points (95% CI 5.0, 13.7) when replacing usual snacks with 57 g pecans (pre-portioned raw, unsalted pecans) daily for 12 weeks [51]. Adding 30 g of pecans to a healthy diet further improved the quality in controlled diets of people with established but stable coronary artery disease [52]. Others have reported improvements in healthy fats, insoluble fiber intake, and magnesium, all nutrients that support higher quality and healthy dietary patterns [53]. Compared to national averages for the diets of people over 2 years of age [50], these studies reveal that replacing components of the diet, like snack foods, with pecans is an easy way to improve diet quality. However, even within a healthy dietary composition, energy balance is critical for maintaining healthy body weight.

## 7. Pecans in Body Weight and Appetite Management

Excess adiposity, or obesity, is a major risk factor for chronic disease development. Obesity is typically characterized by chronic low-grade systemic inflammation, insulin resistance, dyslipidemia, oxidative stress, and hyperglycemia, all of which contribute to progressive tissue and organ dysfunction. These metabolic disturbances elevate the risk for type 2 diabetes mellitus (T2DM), cardiovascular diseases (CVD), and neurodegenerative disorders, including Alzheimer’s disease [54,55]. Weight loss and diet modification are among the most consistent advice for promoting health and reducing disease risk. Foods high in fat or energy density are carefully considered in weight control diets, and as such, nuts, including pecans, are assessed to understand their whole-body benefits relative to their influence on body weight regulation.

### 7.1. Body Weight

Several research studies testing pecan intake on health outcomes have monitored body weight as a secondary endpoint [51,52,53,56,57], and one study examined body weight and composition as a primary outcome [58] (Table 3). Guarneiri and colleagues, (2022) [58] tested the effect of pecan intake (68 g/d) on body weight a priori in sedentary adults (BMI ≥ 28 kg/m^2^, n = 93) for 8 weeks. In this study, pecans were added onto usual intake (ADD) or substituted (SUB) for isocaloric foods in the participant’s usual diet, or followed the usual diet avoiding nuts (Control). Authors reported increases in body weight in all diet groups after 8 weeks and a trend for more body weight gain in the ADD and SUB diets compared to the control (1.1 ± 0.2 kg, 0.9 ± 0.3 kg, 0.3 ± 0.2 kg, respectively, *p* = 0.09). Body composition analysis indicated an increase in body fat in the SUB group (1.0 ± 0.3%; *p* = 0.005) but not the ADD (0.1 ± 0.2%) or control group (−0.2 ± 0.3%). The authors evaluated theoretical weight gain vs. actual and found a large difference between the two with the actual much lower than the theoretical (actual: 1.1 ± 0.2 and 0.9 ± 0.3 vs. theoretical: 3.3 ± 0.0 and 3.2 ± 0.0 kg in ADD and SUB, respectively; *p* < 0.001). In four other studies examining body weight changes as a secondary endpoint, one showed that 12 weeks of pecan consumption (57 g/day) in people with ≥1 criterion for metabolic syndrome (n = 138) tended to increase body weight [51], and three other studies reported no change in body fat [56] or body weight after 4 weeks of intake [56,57], 8 weeks of daily intake [53], or 12 weeks of daily intake [52], ranging from 30 to 68 g pecans per day intake or 20% energy substitution in a Step 1 diet. Overall, the data did not indicate an increased risk of overweight/obesity with pecan intake and average changes were within the daily variability of 1 kg, and consistent with meta-analyses of other nut studies [59,60].

### 7.2. Compensatory Mechanisms

Several reasons may explain why nuts do not pose a risk to weight gain. Adjustment in energy expenditure is one possibility. A systematic review of nuts indicated a non-significant increase in energy expenditure with nut intake [61]; however, pecans may differ in this regard. An increase in select measures of energy expenditure and fat oxidation was reported in an 8-week pre- vs. post-intervention comparison with pecan intake [62]. Another mechanism is the lower metabolizable energy of pecans, which has been documented previously for nuts [63]. Another reason is the behavioral compensation in food intake, influencing total energy intake. The satiety value of foods plays a critical role in compensatory behavior. Five studies investigated subjective and objective changes in satiety and food intake regulation with pecan consumption [56,64,65,66,67]. Subjective satiety was measured by visual analog scales where participants rated their hunger, fullness, desire to eat, and prospective consumption levels. Objective measures were actual food intake after a test load and/or changes in satiety or appetite signaling hormones. In the ADD or SUB protocol described previously, adults with CVD risk factors (n = 47) consumed pecans (68 g) added to their diet (ADD) or substituted (SUB) in their diet for 8 weeks and were tested pre- and post-intervention for changes in acute satiety and appetite measures using a breakfast drink (Ensure ^®^, Abbott, Abbott Park, IL, USA) preload with no nuts [66]. The authors reported greater decreases in prospective consumption and desire to eat in the ADD vs. SUB (−79 ± 41 vs. 11 ± 26 mm/9 h; *p* = 0.05) and ADD vs. control (−64 ± 39 vs. 23 ± 29 mm/9 h; *p* = 0.05), respectively. There was also a non-significant tendency for a greater decrease in overall appetite in the ADD vs. control condition (−67 ± 46 vs. 20 ± 27 mm/9 h; *p* = 0.06). These data were supported by increased postprandial cholecystokinin (CCK) and peptide PYY (PYY) and suppressed postprandial ghrelin [66]. These data indicate that adding pecans to the daily diet provides latent effects in enhancing subjective and physiological markers of postprandial satiety in sedentary adults. Other evaluations investigating post-meal satiety and satiety peptide responses suggested that satiety was not influenced differently by pecan intake after 4 weeks in response to a high fat meal, nor was energy intake, CCK, or ghrelin [56]. However, this group reported a significant increase in PYY in the pecan group compared to the control group. Similarly, satiety after pecans (250 kcal) was not different from a 250 kcal tortilla chip snack, although PYY and glucagon-like peptide-1 (GLP-1) were higher after the pecan vs. tortilla chip snack after 2 h [64]. Another study in healthy young adults reported increased fullness after a meal (795 kcal) containing 68 g pecans (~470 kcal) compared to an isocaloric (795 kcal) control breakfast meal/shake without pecans [65]. Increased PYY and suppressed ghrelin were also reported after the breakfast meal with pecans, along with a decreased appetite at home over the next 780 min. A study by Marquardt (2019) showed reduced satiety in females after consuming a muffin containing pecans compared to a control muffin without pecans [67].

Overall, the data are intriguing, with pecan-associated satiety peptide changes and some reports of enhanced subjective satiety with pecan intake. This is an area worth follow-up, especially in an era of GLP-1 agonist therapy and renewed interest in appetite regulation.

**Table 3:** Diet quality, obesity, satiety.

Ref. No.	First Author/Date	Study Model	Methods, Generally	Duration/Intervention	Results
[51]	Hart T, 2025	Human RCT Parallel 2 Arm	Adults with ≥1 criterion for metabolic syndrome (n = 138) FMD, BP, cf-PWV, lipids/lipoproteins, and glycemic control	12-week intervention Pecans—57 g/d to replace usual snack or Control—usual diet without pecans	↑ Healthy Eating Index ↑ BW (trend) ↓ TC, ↓ LDLc, ↓ non-HDLc, ↓ TG ↔ FMD, ↔ PWV, ↔ BP
[52]	Dos Santos J, 2022	Human RCT Parallel 3 Arm	CAD participants (n = 204) TyG index Markers of glycemic profile and non-traditional anthropometric measures Diet quality: HEI	12-week intervention Pecans—30 g/d in healthy diet (n = 68) or Olive oil—30 mL/d in healthy diet (n = 69) or Control—healthy diet (n = 67)	↑ Healthy Eating Index ↔ Anthropometric measures: body weight, BMI and waist circumference ↔ TyG index (within pecan trend) ↔ Glycemia, ↔ HBA1c, ↔ HOMA-IR
[53]	Morgan W, 2000	Human RCT parallel 2 Arm	Adults with normal lipid levels (n = 19) Blood lipid changes Dietary intake	8-week intervention Pecans—68 g/d or Control—no nuts, self-selected diet	↑ Energy/kcal, ↔ body weight ↓ TC, ↓ HDLc ↓ LDLc (within group) Dietary intake Pecan group ↑ Dietary fat, ↑ monounsaturated fat ↑ Polyunsaturated fat, ↑ insoluble fiber ↑ Magnesium
[56]	Cogan B, 2023a	Human RCT Parallel 2 Arm	Healthy older adults (n = 44) BW and body fat % Subjective appetite—VAS Changes in physiological appetite Energy intake (EI) in-lab and at-home	4-week intervention Pecans—68 g/d (n = 21) or Control—no nuts (n = 23) Pre-and post-4 h postprandial with high fat meal	↔ Body weight, ↔ body fat ↔ Subjective appetite ↓ Peak desire to eat with pecans ↑ PYY, ↔ CCK, ↔ ghrelin ↔ Energy intake (overall) (trend for difference in EI at buffet meal, ↓ pecans vs. ↑ control, *p* = 0.11)
[57]	Rajaram S, 2001	Human RCT Crossover 2 Arm	Healthy adults with elevated lipids (n = 23) Lipid and lipoprotein profiles	4-week intervention Pecans 20% kcal SUB in Step 1 diet (fat energy 39.6%) or Step 1 diet (fat energy 28.3%)	↔ Body weight ↓ TC, ↓ LDLc, ↑ HDL, ↓ TG ↓ Apo B, ↓ Lipoprotein(a), ↑ Apo A1
[58]	Guarneiri L, 2022a	Human RCT Parallel 3 Arm	Adults with overweight or obesity or hypercholesterolemia (n = 93) BW and total body fat %: actual and theoretical changes determined	8-week intervention ADD—68 g/d pecans added to a free-living diet (n = 30) or SUB—68 g/d pecans substituted for isocaloric foods from habitual diet (n = 31) or Control-usual diet no nuts (n = 32)	↑ Body weight (trend ADD, SUB vs. control) ↑ Body fat (SUB) ↔ Body fat (ADD) Actual vs. theoretic changes in BW < in pecan groups (*p* < 0.001) Actual vs. theoretical changes in body fat % < in ADD but not SUB groups (*p* < 0.05)
[62]	Guarneiri L, 2021b	Human RCT Parallel 3 Arm	Adults with overweight or obesity or hypercholesterolemia (n = 52) Indirect calorimetry: resting metabolic rate (RMR) Diet-induced thermogenesis (DIT) and substrate utilization	8-week intervention ADD—68 g/d pecans added to a free-living diet (n = 16) or SUB—68 g/d pecans substituted for isocaloric foods from habitual diet (n = 18) or Control—usual diet, no nuts (n = 18)	SUB group ↑ Fasting RMR, ↑ fat oxidation ↓ Respiratory exchange ratio ADD group ↑ DIT No between-group differences
[64]	Peters J, 2024	Human RCT Crossover 2 Arm	Adults with overweight or obesity (n = 20) Subjective appetite scales, blood markers, and energy expenditure	Pecan (250 kcal) or Control—tortilla chips (250 kcal)	↔ Subjective satiety ↔ Energy intake ↑ PYY ↑ GLP-1
[65]	Prater M, 2024	Human RCT Crossover 2 Arm	Healthy adults (n = 31) Postprandial appetite and blood markers VAS and food records	Postprandial evaluation Pecan (ground)—68 g breakfast shake or Control meal—breakfast shake, macronutrient and calorie matched	↑ Fullness, ↔ hunger, ↔ desire ↔ Prospective consumption, ↓ appetite at home (240–780 min post-meal) Postprandial—↑ PYY, ↓ ghrelin (120 min) ↔ Energy intake
[66]	Guarneiri L, 2022b	Human RCT Parallel 3 Arm	Adults with overweight or obesity or hypercholesterolemia (n = 47) Changes in CCK, PYY, ghrelin, and subjective appetite VAS questionnaires and recorded intake	8-week intervention ADD—68 g/d pecans added to a free-living diet (n = 15) or SUB—68 g/d pecans substituted for isocaloric foods from habitual diet (n = 16) or Control—usual diet, no nuts (n = 16)	ADD > SUB and ADD > Control ↓ Prospective consumption, ↓ desire to eat within ADD ↓ Prospective consumption, ↓ desire to eat, ↑ fullness within ADD Postprandial ↑ CCK, ↑ PYY, ↓ ghrelin ADD > Control ↓ Overall appetite (trend *p* = 0.06) ↔ Energy intake
[67]	Marquardt A, 2019	Human RCT Crossover 2 Arm	Healthy adults (n = 22) TG, antioxidant measures, and VAS for appetite ratings	High saturated fat postprandial test (3 h) Muffin with 28 g pecans (partial replacement for butter) or Control muffin with butter (no pecans)	↔ Satiety, (males) ↓ Satiety (females) ↓ TG, ↓ lipid peroxidation (males) ↑ Total antioxidant capacity (males)

Arrows: ↓ (decrease), ↑ (increase), ↔ (no effect). ADD: Addition group; Apo A1: Apolipoprotein A1; Apo B: Apolipoprotein B; BMI: Body mass index; BP: Blood pressure; BW: Body weight; CAD: Carotid artery disease; CCK: Cholecystokinin; cf-PWV: Carotid–femoral pulse wave velocity; DIT, Diet-induced thermogenesis; EI: Energy intake; FMD: Flow-mediated dilation; GLP-1: Glucagon-like peptide-1; HbA1c: Glycated hemoglobin; HDLc: High-density lipoprotein cholesterol; HEI: Healthy Eating Index; HOMA-IR: Homeostatic Model Assessment of Insulin Resistance; LDLc: Low-density lipoprotein cholesterol; non-HDLc: Non-high-density lipoprotein cholesterol; PYY: Peptide YY; PWV: Pulse wave velocity; RCT: Randomized controlled trial; RMR: Resting metabolic rate; SUB: Substitution group; TC: Total cholesterol; TG: Triglycerides; TyG index: Triglyceride–glucose index; VAS: Visual analog scale.

## 8. Pecans in Cardiometabolic Health: Diabetes and Cardiovascular Disease Risk

### 8.1. Metabolic Health/Diabetes Risk

The growing prevalence of non-communicable diseases is a global public health concern with a significant burden arising from the global increase in metabolic diseases [68]. Metabolic diseases refer to varied metabolic dysregulation processes affecting obesity-linked insulin resistance, glucose homeostasis, lipid metabolism, oxidative stress, chronic low-grade inflammation, and endothelial function. T2DM is one of the most common metabolic diseases with a rapidly increasing incidence and associated mortality [68]. The consumption of nuts and their inclusion in dietary patterns have been associated with beneficial health outcomes; however, specific recommendations related to nuts in diabetes management have been limited.

### 8.2. Glycemic Control

Work specifically with pecans includes four publications on glycemic and/or gluco-regulatory measurements in humans [52,69,70,71], one in vitro investigation reporting on the enzyme inhibiting capability of a pecan extract on starch digestion [72], and an in vivo animal study investigating pecans and pecan polyphenols fractions on varied metabolic outcomes in a diet-induced obesity mouse model [73] (Table 4). The human data reveal inconsistent results on gluco-regulatory endpoints. McKay et al. (2018) fed people who were overweight or had obesity (n = 26) either pecan nuts (~42.5 g) or a fiber/calorie matched control for 4 weeks and reported no difference between the groups on fasting glucose, while insulin concentrations, insulin resistance (HOMA-IR), and beta cell function (HOMA-β) were significantly improved compared to the control diet [69]. In contrast, daily pecan consumption (68 g) for 4 weeks in healthy older adults (n = 41, age 50–75 years) produced neutral effects on fasting and postprandial glucose and insulin between the pecan versus no nut control diet [71]. Similar findings were reported after a parallel 12-week, randomized controlled study with adults with established vascular disease [52]. However, Guarneiri et al. (2021) reported lowered postprandial glucose after an 8-week intake of pecans (68 g) when substituted (SUB) in the diet for isocaloric foods compared to eating no nuts or adding (ADD) pecans to their diet [70]. No changes were observed with fasting insulin, glucose, or postprandial insulin concentrations.

Despite the inconsistent results in human studies, preclinical data suggest that pecan polyphenols potently inhibit α-amylase and α-glucosidase [72]. These data could explain lower postprandial glucose when pecans were SUB for isocaloric food items [70]; however, this could also be due to the substitution strategy in this study since adding pecans (ADD) did not influence postprandial glycemia. Limited preclinical animal data provide mechanistic data for improvements in the metabolic abnormalities of obese mice that may be useful in designing future human studies [73]. Feeding whole/ground pecan or pecan polyphenol extract with a high fat diet reduced fat mass, serum cholesterol, insulin, and insulin resistance (HOMA-IR) by 44, 40, 74, and 91%, respectively, compared to the high fat fed group. In addition, the pecan interventions improved glucose tolerance, prevented pancreatic islet hypertrophy, increased mitochondrial activity and AMPK activation in skeletal muscle, and increased oxygen consumption compared to the high fat diet group. The authors’ conversion to a daily human intake dose is equivalent to ~110–183 g pecan kernels/day (or 22–38 whole pecans) or 21.6–36 g defatted pecan flour/day for an average person of 60 kg. This amount is more than the human studies have tested thus far; however, the data are insightful for designing future human studies, which may include a classic dose response design.

## 9. Cardiovascular Health/Vascular Disease Risk

Cardiovascular diseases account for approximately 17.5 million deaths per year, representing 31% of all deaths globally. Obesity and diabetes contribute significantly to CVD risk. Diabetes increases the risk of a cardiovascular event by 3–4 times [83]. Therefore, achieving and maintaining a healthy body weight and managing cardio-metabolic risk factors is the top priority for reducing the risk of a cardiac event. Dietary guidelines and clinical practice guidelines recommend the importance of a healthy dietary pattern with consumption of whole grains, fruits and vegetables, legumes, fish, and nuts, aiming to reduce the intake of unhealthy fats, such as saturated and trans fatty acids, to manage the lipid profile and prevent CVD. In addition to a healthy fatty acid composition, nuts contain fiber and polyphenols with potential heart health benefits. Systematic reviews and meta-analyses have demonstrated that nut consumption decreases TC and LDL levels, as well as TG [60]. The effects of pecans on lipid metabolism and other risk factors of CVD are discussed below.

### 9.1. Pecans and Lipid Metabolism

Published data from 2000 to 2025 have reported that pecan intake reduces TC, LDL, and TG and, when calculated, reduces non-high density lipoprotein cholesterol (non-HDL cholesterol) concentrations (Table 3: [51,52,53,57,67], Table 4: [59,69,74,75,76,77]). A single-blinded randomized controlled trial conducted by Hart et al. (2025) reported that consuming 57 g/day of pecans for 12 weeks significantly decreased TC (~8.1 mg/dL), LDLc (~7.2 mg/dL), non-HDLc (~9.5 mg/dL), and TG (~16.4 mg/dL) compared to a control diet in adults with ≥1 criterion for metabolic syndrome who were free from CVD and T2DM (n = 138) [51]. Another study conducted on older adults (59 ± 6 years) showed that 4 weeks of consuming 68 g/day of pecans significantly reduced the pre- to post-diet TC (*p* = 0.04), LDLc (*p* = 0.01), non-HDLc (*p* = 0.02), LDL particles (*p* = 0.01), and medium size LDL particles (*p* = 0.01) compared to a control group [74]. Furthermore, when dynamic state lipid responses were tested, postprandial TG was lowered after the pecan diet (*p* = 0.01) compared to after the control diet (*p* = 0.78). Lipid lowering effects were also reported by Guarneiri et al. (2021) [59] who tested the effects of adding pecans to the diet (ADD) or substituting (SUB) them for other foods in the diet (see Table 3, Body weight section). The results showed a significant within-group reduction after 8 weeks of pecan intake on TC, LDLc, TG, TC/HDL ratio, non–HDL cholesterol, and apolipoprotein B in both the ADD and SUB groups (*p* ≤ 0.05 for all), while no changes were observed in the control group [59]. Postprandial TG was also reduced within the ADD group (*p* ≤ 0.01). Similarly, earlier work showed that replacing 20% of total energy intake with pecans to increase monounsaturated fat intake significantly lowered TC, LDLc, and TG, while increasing HDLc (*p* < 0.05, all) after 4 weeks compared to the Step 1 diet [57]. Pecan consumption also significantly reduced serum apolipoprotein B (−11.6%) and lipoprotein(a) (−11.1%), while increasing apolipoprotein A1 (+2.2%) compared to the Step 1 diet (all *p* < 0.05). A study in adults with normal lipid levels (n = 19) demonstrated that participants assigned to a pecan group (68 g/day) for 8 weeks and to a nut-free control group decreased LDLc significantly in the pecan group by week 4 (2.61 → 2.35 mmol/L) and remained lower at week 8 (2.46 mmol/L, *p* < 0.05). Furthermore, in week 8, total cholesterol and HDLc levels were significantly lower in the pecan group than in the control group (*p* < 0.05) [53]. In healthy overweight or obese individuals with central adiposity, consuming 15% of the total daily calories from pecans (~42.5 g/2000 Kcal) for 4 weeks lowered both TC and LDLc, although the magnitude of change compared with the control diet (typical American diet) was marginally significant (*p* = 0.056, *p* = 0.067, respectively). In a randomized controlled trial by Campos et al. (2020), 204 patients with stable coronary artery disease were assigned to consume either 30 g of pecans per day, 30 mL of olive oil per day, or a healthy diet alone (control) for 12 weeks [77]. Compared with the control and olive oil groups, the pecan group showed a significant reduction in non-HDL cholesterol (*p* = 0.033) and in the TC/HDL cholesterol ratio (*p* = 0.044) [77]. Another publication by the same group (n = 204) showed 30 g/day of pecan intake marginally influenced the triglyceride–glucose (TyG) index, considering an intra-group analysis (−0.1495% CI −0.28 to 0.0008; *p* = 0.053). However, the final TyG indices were not significantly different between the healthy diet (control group) and the 30 mL/day of extra virgin olive oil groups. There were no differences in the relation to all other lipid parameters evaluated [52]. Few preclinical data are available, but an animal study with C57BL/6 mice demonstrated that supplementation of a high fat (HF) diet with 30% whole pecan or pecan polyphenols (3.6 or 6 mg/g) for 18 weeks significantly reduced serum TC by 44% compared to the HF diet alone [73]. 

### 9.2. Pecans and Emerging CVD Risk Factors

Apart from traditional CVD-related markers, such as blood lipids, non-traditional risk factors, including measures of endothelial dysfunction, oxidative stress markers, and chronic low-grade inflammation, have been investigated as targets for improvement with pecan consumption due to their involvement in early and intermediary disease pathology. Although studies have reported measurements of endothelial function, no clear evidence is available, likely due to the limitations of the study models. Hart et al. (2025) reported no significant effects on endothelial function, as measured by flow-mediated dilation (FMD), blood pressure, or carotid–femoral pulse wave velocity (cf-PWV) [51]. Consistent with these findings, FMD and reactive hyperemia did not differ from the control after pecan intake; however, the postprandial reactive hyperemia slope and reactive hyperemia time to half significantly differed by group, shown by improvements in the pecan group compared to the control [74]. Increased oxidative stress and inflammation are known to be associated with several chronic disease developments, including CVD. Several papers have been published describing the antioxidant activity associated with pecan intake in humans [47,74,78,79] or animals [80]. Pecan intake has been shown to increase antioxidant capacity by 12% and 10% at 2 h, respectively, as measured by hydrophilic and lipophilic ORAC assays as well as reduce oxidized LDL. Similarly, measures of lipid oxidation have suggested a protective role by pecans when consumed acutely or regularly for 4 weeks in younger and older individuals [47,74,78,81].

Although several studies showed that daily pecan consumption protects against oxidative stress and markers associated with CVD, few studies have investigated the effect of pecan consumption on inflammatory markers [69,76,77]. Weschenfelder et al. (2022) reported no significant effect on the inflammatory markers by including pecan nuts (30 g/d of pecans + healthy diet) or extra virgin olive oil (30 mL/d of extra virgin olive oil + healthy diet) compared to a healthy diet on the inflammatory markers in individuals with coronary artery disease (n = 204) [76]. An earlier publication with patients with stable coronary artery disease also found that, after adjusting for sex, statin use, and changes in body weight, no between-group differences in inflammatory markers were observed; however, interleukin-6 (primary outcome) decreased significantly in all groups: control, pecan, and olive oil compared with baseline after 12 weeks of intervention [77]. Other emerging markers have included measuring angiopoietin-like proteins (ANGPTLs). These proteins are known for their roles in lipid and glucose metabolism, inflammation, hematopoiesis, and cancer [84]. Two papers by Guarneiri et al. (2022) and (2021) [79,82] showed that adding and substituting pecans in the diet reduced postprandial ANGPTL3 levels in healthy adults from pre- to post-intervention (*p* < 0.05) while the control group showed no significant change (*p* > 0.05). A secondary analysis suggested that ANGPTL3 was suppressed more in males (*p* < 0.05) who consumed pecans (68 g) compared to the control group [82]. Other ANGPLTs (-4 and -8) did not change [79,82]. ANGPTL3 is known to play a role in enhancing lipid metabolism.

Overall, human and animal research data from 2000 to 2025 show consistent reductions in TC, LDLc, TG, and non-HDLc with pecan consumption, along with improvements in apolipoproteins and postprandial lipid metabolism. Antioxidant defenses and reductions in lipid peroxidation have also been documented. Results on vascular function and inflammation are inconsistent, suggesting that pecans exert their strongest effects on lipid metabolism and oxidative stress, while their impact on other CVD risk-related markers requires further research.

## 10. Pecans in Brain and Gut Health

### 10.1. Brain Health

More than 55 million people worldwide are living with dementia [85]. Alzheimer’s disease (AD) is the most common form of dementia and is one of the most widely researched areas today. Excessive oxidants (ROS: reactive oxygen species) and inflammatory entities are considered to be at the root of AD development. Pecans are a rich source of fiber, several micronutrients, and an array of polyphenol compounds with demonstrated antioxidant and anti-inflammatory properties. Selected polyphenol metabolites cross the blood–brain barrier and may influence oxidative stress, inflammation, and other signaling pathways important for disease prevention [86].

During the last couple of decades, fruits, particularly berries, have been investigated for their effects on cognitive function. Pecans share some of the same types of polyphenols that berries contain, which may have effects on brain function, specifically proanthocyanidins and anthocyanins. A proanthocyanidin-rich diet can be neuroprotective and have a positive effect on reducing the risk for Alzheimer’s disease as well as other neurodegenerative diseases like Parkinson’s disease, which can also be a cause of dementia [87,88]. Mechanisms of action are likely both direct and indirect, influencing processes managing oxidative stress and damage, inflammation, cholinergic modulation, amyloid beta precursor processing and microglial activation, preservation of the brain-derived neurotrophic factor (BDNF), and possibly other preventative actions [89]. However, further research is required to bridge the gap between preclinical evidence and clinical application. Work in pecans considers the whole nut, which includes valuable polyphenols, but also work with fractions, such as phospholipids, that may play a role in brain health.

Two studies in healthy older adults investigated the effects of pecan intake acutely and after 4 weeks of intake [90,91] (Table 5). In the acute setting, a pecan-enriched shake providing 68 g of pecans, approximately 470 kcals from pecans, improved cognitive performance on four attention and processing speed tests and four memory and learning tests, whereas the isocaloric control shake improved performance on three of twenty-three measures over 4 h post-shake intake [90]. In the 4-week trial, cognitive function was assessed using a fluid composite score and four subtests from the NIH Toolbox Cognitive Battery (NIHTB-CB). No differences were detected in cognitive performance measures between diets without nuts and those incorporating pecans (68 g) in the diet daily [91]. Jia et al. (2025) published their work on extracts of pecans, specifically investigating the phospholipid fraction at different doses (30–120 mg/kg) [92]. The control group received 200 or 400 mg/kg of soy phospholipids. Their work in a mouse model with dementia revealed improvements in learning and memory domains in step-through and step-down tests and the Morris water maze [92]. Overall, the data are limited and provide insight for future studies. Likewise, the neutral effects in a generally healthy older adult cohort [91] should not discourage investing further in this space, as the results may not have been unexpected given the duration of the trial. Researchers studying berries and grape seed extract and other proanthocyanidin rich plant foods typically examine cognitive outcomes after a minimum of 8 weeks, and more often after 12 or 16 weeks of intervention. Additionally, conducting studies in individuals with self-described memory challenges may provide a better opportunity to measure a benefit in people.

### 10.2. Gut Health

Gut health and the role of the gut microbiome in health and disease risk have been a center point for research investigations since early studies showed the association between dysbiosis and obesity, and then later with several chronic diseases [93,94]. The microbial community and its symbiotic relationship with the host (humans in this case) is designed to produce an ecological environment where both thrive. However, environmental exposures, of which diet is one of the main exposures, influence relationships and consequential (whether positive or negative) health outcomes. Pecans contain many constituents that would serve as substrates for the human microbiome to generate bioactive metabolites for possible health benefits.

The effects of pecans on gut health through alterations in gut microbiome are limited. We found one mouse model study investigating pecans or pecan polyphenol fractions on microbial endpoints (Table 5). Mice were divided into five groups and fed standard control diets or high fat diets, or a high fat diet with 30% of energy from pecans or supplemented with 3.6 and 6.0 mg/kg of pecan polyphenols daily for 4 weeks. Authors evaluated fecal microbial diversity and found that the mice fed diets with whole pecans or the 6 mg phenolic extract had increased observed species and richness. They also investigated whether differences in microbiota diversity would reduce circulating LPS (a pro-inflammatory mediator and indicator of gut permeability). LPS was significantly lower in the pecan and pecan polyphenols groups compared to the high fat fed diet, and similar to the control low fat fed group [73]. The authors concluded that pecan phenolics are involved in shaping intestinal microbial communities and maintaining eubiosis despite the elevated fat content in the diet. Work with other tree nuts has not been conclusive. Creedon et al. (2020) reviewed the available literature and found the strength of evidence from the meta-analyses weak [95]. They suggested that future parallel design randomized controlled trials, powered to detect changes in primary outcomes related to the gut microbiota and incorporating clinical and functional outcomes, were necessary to gain robust conclusions on the impact of nuts on the gut microbiota and gut health [95]. Research on the effects of pecans in human studies would help expand our knowledge. Including functional readouts linked to metabolites generated by microbes would also lend insight into biological activity and the benefits of pecans to human health.

## 11. Summary and Future Work

The purpose of this literature review is to summarize the research from the past 25 years on the health benefits of pecans. While we have highlighted the key strengths and limitations of existing studies under each section, a more detailed examination of publication bias, specific methodologies, and outcomes is warranted, but falls beyond the scope of this review paper. Human trials indicate consistent improvements in lipid profile and overall diet quality when pecans replace typical snacks or foods, with largely neutral effects on body weight and inconsistent effects on glycemic control, vascular function, and inflammation. The variations in human clinical trials could be due to differences in pecan doses or forms used, study duration, study design, and population studied. Future clinical studies should be designed with these factors in mind.

Pecans have high (poly)phenol content; however, there is limited information on their bioavailability and metabolic fate following consumption. Future work should characterize the absorption and metabolism of major pecan (poly)phenols and their metabolites. Both short-term (one-time intake) and long-term (regular intake for a month or more) interventions would be ideal for capturing differences in microbial-derived metabolites that may increase/change due to the modified composition and function of the gut microbiome. Information on the cardiometabolic benefits of pecans in well-controlled human studies is available, particularly in lipid management, but limited in many areas, such as CVD risk, specifically vascular function, diabetes risk, and metabolic health (emphasizing insulin sensitivity and inflammation research). More research is warranted in emerging areas of brain and gut health. Research on satiety outcomes is worth following up, particularly in an era of renewed interest in appetite regulation and the unmet needs for appropriate dietary regimens to pair with pharmaceutical therapies (i.e., GLP-1 agonists).

Overall, the current literature suggests that pecans offer cardiometabolic benefits and enhance diet quality when incorporated into healthy dietary patterns; however, further research is needed to expand our understanding of other clinical outcomes, especially related to mechanistic pathways and their role in reducing the risk of chronic diseases.

## Figures and Tables

**Table 1:** Nutritional content of raw pecan nuts.

Nutrient/Compound	Average Amount (Per 100 g)
Calories (kcal)	700
Protein (g)	9.96
Ash (g)	1.44
Total fat (g)	73.3
Fatty acids, total saturated (g)	6.46
Fatty acids, total monounsaturated (g)	39.3
Fatty acids, total polyunsaturated (g)	22.9
Carbohydrates	
Carbohydrates by difference (g)	12.7
Fiber, total dietary (g)	5.8
**Minerals**	
Calcium (mg)	55
Iron (mg)	2.37
Magnesium (mg)	103
Phosphorus (mg)	253
Potassium (mg)	360
Sodium (mg)	<2.5
Zinc (mg)	3.93
Copper (mg)	0.91
Manganese (mg)	2.28
Molybdenum (µg)	15.6
**Vitamins**	
Thiamin (mg)	0.55
Niacin (mg)	0.95
Vitamin B-6 (mg)	0.17
Biotin (µg)	22.7
Vitamin K (phylloquinone) (µg)	4.1
Vitamin K (Dihydrophylloquinone) (µg)	<0.1
Vitamin K (Menaquinone-4) (µg)	<0.1
**Amino Acids**	
Tryptophan (g)	0.115
Threonine (g)	0.302
Isoleucine (g)	0.365
Leucine (g)	0.67
Lysine (g)	0.32
Methionine (g)	0.168
Phenylalanine (g)	0.462
Tyrosine (g)	0.312
Valine (g)	0.435
Arginine (g)	1.36
Histidine (g)	0.262
Alanine (g)	0.438
Aspartic acid (g)	0.87
Glutamic acid (g)	2.5
Glycine (g)	0.492
Proline (g)	0.61
Serine (g)	0.54
Hydroxyproline (g)	<0.01
Cysteine (g)	0.305
**Phytosterols**	
Stigmasterol (mg)	3
Campestrol (mg)	6
β-Sitosterol (mg)	117
**Flavonoids**	
Catechin (mg)	7.24
Cyanidin (mg)	10.74
Delphinidin (mg)	7.28
Epicatechin (mg)	0.82
Epigallocatechin (mg)	5.63
Epigallocatechin gallate (mg)	2.30
**Proanthocyanidins**	
Monomers (mg)	17.22
Dimers (mg)	42.13
Trimers (mg)	26.03
4–6 mers (mg)	101.43
7–10 mers (mg)	84.23
Polymers (mg)	223.01

Sources: National Nutrient Database for Foundation Foods, United States Department of Agriculture, FDC published: 28 October 2022, FDC ID: 2346395, NDB number: 12142. Vitamin K (phylloquinone) and fatty acids, total saturated and total monounsaturated and total polyunsaturated, median values corrected on 18 April 2024. Phytosterol data from USDA Database for Standard Reference Legacy, released April 2018; Database of Flavonoid Values for USDA Food Codes and Flavonoid Intake Data Files from What We Eat in America (WWEIA), National Health and Nutrition Examination Survey (NHANES), released 2017–2018. USDA Database for the Proanthocyanidin Content of Selected Foods, released 2.1—2018.

**Table 2:** Pecan Bio-accessibility and Bioavailability.

Ref. No.	First Author Date	Model	Methods, Generally	Interventions	Results
[46]	Cheung M, 2023	In Vitro Digestion Model	Acetonic crude extracts: raw and roasted pecans Digested and undigested extracts of raw pecan phenolics were separated into low- and high-molecular-weight (LMW and HMW) fractions via Sephadex LH-20 column chromatography Characterization: RP-HPLC–ESI–MS	Raw and roasted Georgia pecans	LMW fraction: flavan-3-ols and ellagic acid derivatives. Post-digestion: overall ↓ phenolics from 16% to 100% HMW fraction: procyanidins with DP 2–6 Post-digestion: ↓ higher oligomeric procyanidins and significant ↑ in quantity of dimers. ↓ in antioxidant capacity ↓ TPC was mirrored by the ↓ in overall phenolics, as evidenced via HPLC analysis HPLC–ESI–MS/MS: digestion altered the phenolic profile of the LMW and HMW fractions ↑ Dimers from depolymerization (DP4–6) and dimerization of catechin/epicatechin
[47]	Hudthagosol C, 2011	Human RCT Crossover 3 Arm	Healthy men and women (n = 16)	Postprandial study (24 h) pecans, 90 g whole or blended or isocalorie control meal	↑ γ-tocopherols (8 h) ↑ Hydrophilic- and lipophilic-ORAC (2 h) ↑ Epigallocatechin-3-gallate concentrations (1 and 2 h) ↓ Oxidized LDL (whole: 2, 3, 8 h) ↓ MDA:TG (pooled pecan data 3, 5, 8 h)
[48]	Kang M, 2023	Human RCT parallel 2 Arm	Adults with overweight or obesity or hypercholesterolemia (n = 61)	4-week intervention ADD—68 g/d pecans added to a free-living diet (n = 16) or SUB—68 g/d pecans substituted for isocaloric foods from habitual diet (n = 20) or Control—usual diet, no nuts (n = 25)	↑ Urolithins (A and C) and ellagic acid after pecan intake Urolithin A glucuronide was most abundant after 4 weeks of pecan intake (2.6–106 ng/mL)

Arrows: ↓ (decrease), ↑ (increase). DP: Degree of polymerization; ESI–MS/MS: Electrospray ionization tandem mass spectrometry; HMW: High-molecular-weight; HPLC: High-performance liquid chromatography; LDL: Low-density lipoprotein; LMW: Low-molecular-weight; MDA: Malondialdehyde; MDA:TG: Malondialdehyde-to-triacylglycerol ratio; ORAC: Oxygen radical absorbance capacity; RCT: Randomized controlled trial; TPC: Total phenolic content.

**Table 4:** Cardiometabolic health: metabolic/diabetes and cardiovascular, lipids, inflammation.

Ref. No.	First Author Date	Study Design	Methods, Generally	Duration/Interventions	Results
			**Metabolic/Diabetes**		
[69]	McKay D, 2018	Human RCT Parallel 2 Arm	Overweight or obese adults with central adiposity (n = 26) Biomarkers related to CVD and T2DM risk	4-week intervention Pecan diet: Control diet with 15% total kcal replaced by pecans Control diet: Isocaloric diet matched for fiber and fat (typical American diet)	↓ Insulin * ↔ Glucose ↓ HOMA-IR *, ↓ HOMA-β ↓ TC (trend), ↔ VLDL ↓ LDL (trend), ↔ HDL ↔ TG, ↔ Oxidized LDL ↔ SBP *, ↔ DBP ↔ CRP, ↔, E-selectin, ↔ Endothelin ↔ Plasma phenolics, ↔ tocopherols, * subgroup analysis, the greater the impairment the better the pecan-associated outcomes
[70]	Guarneiri L, 2021c	Human RCT Parallel 3 Arm	Adults at risk for cardiovascular disease Changes in lipid peroxidation and antioxidant capacity	8-week intervention, at pre- and post-intervention: 2 h postprandial effects ADD—68 g/d pecans added to a free-living diet (n = 15) or SUB—68 g/d pecans substituted for isocaloric foods from their habitual diet (n = 16) or Control—usual diet, no nuts (n = 16)	↓ Postprandial lipid peroxidation ↑ Total antioxidant capacity ↑ γ-tocopherol (within groups)
[71]	Cogan B, 2023b	Human RCT Parallel 2 Arm	Older adults (n = 41) Glucoregulatory effects and lipid peroxidation	4-week intervention with 2 h high fat meal challenge pre- and post-intervention Pecans—68 g/d or Control—usual diet without pecans	↔ Insulin, ↔ glucose ↓ Lipid peroxidation (post-meal) ↔ Total antioxidant capacity
[72]	Feng J, 2022	In Vitro	Enzyme kinetic study: α-amylase and α-glucosidase	Pecan extract Phenolic compounds	Pecan extract: inhibited α-amylase, α-glucosidase activity, and starch digestion ↓ Glucose release during simulated digestion
[73]	Delgadillo-Puga C, 2023	Animal Parallel 5 Arm	C57BL/6 mice Glucose/lipid metabolism markers Gut microbes	18-week intervention Control diet (7% fat) or high fat (HF) diet (23% fat) or Whole Pecans 30% energy or Pecan Polyphenols 3.6 or 6.0 mg/g in HF diet	↓ Insulin, ↓ HOMA-IR ↓ Glucose intolerance, ↓ TC, ↑ Mitochondrial activity ↓ Serum LPS ↑ Brown adipose thermogenesis ↑ AMPK activation, ↑ O_2_ consumption ↑ Alpha diversity ↑ Beta diversity ↔ Species enrichment
			**Lipids and emerging risk factors**		
[74]	Cogan B, 2023c	Human RCT Parallel 2 Arm	Healthy older adults (n = 44) Fasting and postprandial lipids and vascular function, FMD, and reactive hyperemia (RH)	4-week intervention Pecans—68/d (n = 21) or Control—usual diet without nuts (n = 23)	↓ TC, ↓ LDLc, ↓ nonHDLc ↓ LDL particle, ↓ LDL particle medium ↓ Postprandial TG ↔ FMD, ↔ RH, ↑ postprandial RH slope ↑ RH time to half
[75]	de Araújo A, 2021	Human RCT Parallel 3 Arm	Patients with stable coronary artery disease (n = 204) Plasma fatty acids (n = 149 included in analysis)	12-week intervention Pecans—30 g/d in a healthy diet or Olive oil—30 mL/d in a healthy diet or Control—healthy diet	↔ Plasma free fatty acids Correlations with lipid profiles weak
[76]	Weschenfelder C, 2022	Human RCT Parallel 3 Arm	Patients with established coronary artery disease (n = 204) Inflammatory markers	12-week intervention Pecans—30 g/d in a healthy diet or Olive oil—30 mL/d in a healthy diet or Control—healthy diet	↔ IL-6, ↔ hsCRP ↔ Fibrinogen, ↔ IL-2 ↔ IL-4, ↔ IL-10 ↔ IFN-γ, ↔ IL-6/IL-10 ↔ IL-2/IL-4, ↔ IFN-/γIL-4
[77]	Campos V, 2020	Human RCT Parallel 3 Arm	Patients with stable coronary artery disease (n = 204) Lipid profile	12-week intervention Pecans—30 g/d in a healthy diet or Olive oil—30 mL/d in a healthy diet or Control—healthy diet	↔ LDLc, ↔ HDLc ↔ LDLc/HDLc, ↔ HDLc/TG ↓ nonHDLc, ↓ TC/HDLc ↓ IL-6 all groups
[78]	Haddad E, 2006	Human RCT Crossover 2 Arm	Healthy adults (n = 24) Tocopherols and measures of antioxidant capacity and oxidative stress	4-week intervention Pecan—20% of energy in a diet or Control—usual diet	↓ Lipid peroxidation by MDA ↑ γ-tocopherol, ↓ α-tocopherol ↔ Antioxidant capacity (FRAC and Trolox)
[79]	Guarneiri L, 2022c	Human RCT Parallel 3 Arm	Adults with overweight or obesity or hypercholesterolemia (n = 47) Determine changes in angiopoietin-like protein (ANGPTL) 3, -8, and -4	8-week intervention ADD—68 g/d pecans added to a free-living diet (n = 15) or SUB—68 g/d pecans substituted for isocaloric foods from their habitual diet (n = 16) or Control—usual diet, no nuts (n = 16)	Postprandial ↓ Angiopoietin-like protein (ANGPTLs)-3, ↔ ANGPTL-8, ↔ ANGPTL-4 (within group changes ADD and SUB)
[80]	Domínguez-Avila J, 2015	Animal Parallel 5 Arm	Male Wistar rats (n = 30) Lipid metabolism and antioxidant defense (i.e., enzyme activities)	9-week intervention Control diet or high fat diet (HFD), or ground pecan (1/2 fat of HFD) or pecan oil (1/2 fat of HFD) or pecan polyphenol fraction (1 mg/g HFD)	Compared to Control HFD: ↑ TC, ↑ leptin Whole pecan: ↓ TC, ↑ Apo B mRNA, ↑ LDL-R mRNA, ↓ lipid peroxidation, ↓ leptin, ↑ catalase, ↑ GPx, ↑ GST Oil: ↓ TG Polyphenols: ↑ Liver X receptor α mRNA
[81]	Guarneiri L, 2021d	Human RCT Parallel 3 Arm	Adults with overweight or obesity or hypercholesterolemia (n = 56) Changes in blood lipids and glycemia	8-week intervention ADD—68 g/d pecans added to a free-living diet (n = 16) or SUB—68 g/d pecans substituted for isocaloric foods from their habitual diet (n = 18) or Control—usual diet, no nuts (n = 18)	Fasting ↔ Insulin, ↔ glucose ↓ TC, ↓ LDLc, ↓ TG, ↓ TC/HDL ↓ nonHDLc, ↓ Apo B (ADD, SUB: within-group comparison) Postprandial ↓ TG (ADD, within group) ↓ Glucose (SUB, within group) ↔ Insulin
[82]	Guarneiri L, 2021e	Human RCT Crossover 2 Arm (Study 1) 3 Arm (Study 2)	A secondary analysis using two studies that were double-blinded RCT Blood was collected at fasting, 30, 60, 120, and 180 min postprandially to determine changes in ANGPTL	Study 1 (n = 22) Pecans, 28 g or No nuts Study 2 (n = 30) Black walnuts or English walnuts or No nuts	In study 1, ↓ ANGPTL3, ↔ ANGPTL-4 In study 2, ↔ ANGPTL3, ↔ ANGPTL-4

Arrows: ↓ (decrease); ↔ (no effect); ↑ (increase). AMPK, 5′ AMP-activated protein kinase; BMI, Body mass index; BW, Body weight; BP, Blood pressure; CCK, Cholecystokinin; FMD, Flow-mediated dilation; GLP-1, Glucagon-like peptide-1; HDL, High density lipoprotein; HOMA-IR, Homeostatic Model Assessment for Insulin Resistance; LPS, Lipopolysaccharide; non-HDL-c, Non-high density lipoprotein cholesterol; PWV, Pulse wave velocity; PYY, Peptide YY; RMR, Resting metabolic rate; TC, Total cholesterol; TG, Triglycerides; RCT, Randomized controlled trial; TyG, Triglyceride–glucose index.

**Table 5:** Brain and gut health.

Ref. No.	First Author/Date	Study Design	Methods, Generally	Duration/Interventions	Results
[90]	Guadagni A, 2025	Human RCT Crossover 2 Arm	Healthy adults (n = 31) A cognitive battery, VAS for motivation, and the Stanford Sleepiness Scale were administered at fasting and 1, 2, 3, and 4 h postprandially	4 h postprandial study Pecan-enriched shake 68 g/d or Control—calorie matched high saturated fat shake	↑ Cognitive performance 8/23 cognitive measures > Pecan 4 attention and processing 4 memory and learning 3/23 cognitive measures > Control
[91]	Cogan B, 2022	Human RCT Parallel 2 Arm	Healthy adults (n = 42) Cognitive function assessments: fluid composite score and four subtests from the NIH Toolbox Cognitive Battery (NIHTB-CB) (Flanker Test, Digital Change Card Sort Test (DCCS), Picture Sequence Memory Test (PSMT), NIHTB Auditory Verbal Learning Test (RAVLT)) at fasting and 30 and 210 min after interventions	4-week intervention Pecans—68 g/d or Control—high saturated fat meal without nuts	↔ Cognitive performance
[92]	Jia X, 2025	Animal Parallel 5 Arm	Demented mice (n = 210) Behavioral tests, including the step-through test, the step-down test, and the Morris water maze test	4-week intervention Pecan phospholipid extract dose: 120, 60, 30 (mg/kg, IG) vs. Soy phospholipids: 400 and 200 (mg/kg, IG) intragastrically administered	↑ Learning, ↑ memory via step-through test, step-down test, and Morris water maze test

Arrows: ↔ (no effect); ↑ (increase).

## Data Availability

No new data were created or analyzed in this study.

## Supplementary Materials

The following supporting information can be downloaded at: https://www.mdpi.com/article/10.3390/nu17233686/s1, Supplementary Table S1: Pecan (poly)phenols.

## Abbreviations

DM	Diabetes mellitus
OW/OB	Overweight and Obese
hs-CRP	Highly sensitive C-reactive protein
CVD	Cardiovascular disease
NHANES	National Health and Nutrition Examination Survey
OGTT	Oral Glucose Tolerance Test
IL-6	Interleukin 6
TNF-α	Tumor Necrotic Factor alfa
HOMA	IR Homeostasis Model Assessment of Insulin Resistance
IGI	Insulinogenic index
AUCs	Area under the curves (AUCs)
LDL	Cholesterol—Low-density Cholesterol
HDL	Cholesterol—High-density Cholesterol
AMPK, 5′AMP	Activated protein kinase
BMI	Body mass index
BW	Body weight
BP	Blood pressure
CCK	Cholecystokinin
FMD	Flow-mediated dilation
GLP-1	Glucagon-like peptide-1
LPS	Lipopolysaccharide
PWV	Pulse wave velocity
PYY	Peptide YY
RMR	Resting metabolic rate
TyG	Triglyceride–glucose index
VAS	Visual analog scale
ADD	Addition group
Apo A1	Apolipoprotein A1
Apo B	Apolipoprotein B
DP	Degree of polymerization
ESI-MS/MS	Electrospray ionization tandem mass spectrometry
HMW	High-molecular-weight
HPLC	High-performance liquid chromatography
LMW	Low-molecular-weight
MDA	Malondialdehyde
ORAC	Oxygen radical absorbance capacity
RCT	Randomized controlled trial
TPC	Total phenolic content
ROS	Reactive oxygen species
