# Supplementary material for: Pecans and Human Health: Distinctive Benefits of an American Nut
Source: Nutrients. 2025 Nov 25;17(23):3686. doi: 10.3390/nu17233686 (PMC12694537; doi:10.3390/nu17233686)
Supplement: Supplementary file 1 [file nutrients-17-03686-s001.zip › nutrients-3966671-supplementary.pdf]

**Supplementary Table S1:** Pecan (poly)phenols

| Author, Year (Ref. No.) | Study type | Study methods/variables | Pecan cultivars/Nut type | Geographical location | Major findings |
|---|---|---|---|---|---|
| Ferrari V, 2022 [14] | Cultivar comparison | Proximate Composition and Minerals<br><br>Fatty Acid Profile (GC-FID)<br><br>TPC<br>Condensed Tannins-Vanillin method<br><br>Tocopherols-HPLC-DAD<br><br>Total Anthocyanins-pH differential<br><br>AOX-ORAC | 16 cultivars | Uruguay | Genetic variability<br>Pecan cultivars in Uruguay \| Significant differences in nutritional & bioactive composition<br><br>Phenolic content<br>Kernels: 18–41 mg GAE/g \| AOX in shells 5x higher than kernels<br><br>Fatty acids<br>High monounsaturated fats \| Oleic acid most abundant<br><br>Tocopherols<br>γ-tocopherol dominant \| Contributes to antioxidant properties |
| Yilmaz R, 2021 [16] | Nut characteristics<br><br>Biochemical composition | Physical traits measured- nut weight, kernel weight, and shell thickness<br><br>Oil content-Soxhlet extraction | 5 pecan cultivars<br><br>Burkett<br>Choctaw<br>Mahan<br>Western<br>Wichita | Turkey | Pecan cultivar differences<br>Nut weight, kernel weight, shell thickness \| Significant variations<br><br>Oil content<br>63.88% - 72.45% \| ‘Mahan’ highest<br><br>Phenolic content<br>Varied by cultivar \| ‘Wichita’ highest |

| | | | | | |
|---|---|---|---|---|---|
| | | TPC<br><br>AOX-DPPH assay<br><br>Fatty acid-GC-FID | | | AOX<br>Positive correlation with phenolic content |
| Woźniak M, 2022 [23] | Nut comparison | Phenolic compounds- HPLC<br><br>AOX-DPPH and ABTS<br><br>Mineral- ICP-OES | 10 types of nuts<br><br>Pecans<br>Walnuts<br>Cashews<br>Macadamia<br>Hazelnuts<br>Almonds<br>Brazil nuts<br>Pistachios<br>Pine nuts<br>Peanuts | Poland | Phenolic content<br>Pecan nuts → Highest among 10 nut types<br><br>Dominant phenolics<br>Gallic acid & protocatechuic acid → Found in pecans<br><br>Antioxidant activity<br>Strong → Linked to high phenolic content<br>Minerals<br>Pecans → Rich in potassium, phosphorus, magnesium, calcium<br><br>Pecans → Nutritionally dense, valuable for antioxidants & minerals |
| Bouali I, 2023 [26] | Ripening stages<br>Cultivars | Phenolic Extraction<br><br>Estimation of TPC, TF, TCT, Total o-diphenols, Total flavaonols<br><br>HPLC-DAD Analysis of Individual Phenolic Compounds | 3 cultivars<br><br>Burkett, Mahan, and Moore | Tunisia | Phenols, flavonoids, tannins<br>↑ in immature pecans; ↓ with ripening<br><br>Ellagic acid & catechin<br>Dominant phenolics; ↓ with maturity<br><br>Antioxidant activity (DPPH, FRAP)<br>↓ as nuts ripened<br><br>Sugars (sucrose, glucose, fructose)<br>↓ initially, then ↑ at full maturity<br><br>Cultivar variation |

| | | | | | |
|---|---|---|---|---|---|
| | | Estimation of Antioxidant Activity (DPPH and FRAP)<br><br>Carbohydrate Analysis (HPLC-RI) | | | Burkett: ↑ early phenolics<br>Mahan: ↑ phenolics at maturity<br><br>Polyphenols, antioxidants & sugars Affected by cultivar and maturity stage. |
| Jia X, 2018 [27] | Phenolics Ripening stages | TPC<br><br>TF<br><br>TCT<br><br>AOX: DPPH and ABTS<br><br>Phenolic compounds were identified using UPLC-Q/TOF-MS,<br><br>Quantification-HPLC-DAD | 5 pecan cultivars<br><br>Pawnee<br>Stuart<br>Wichita<br>Jinhua<br>Shaoxing | China | Phenolic content and AOX in pecan kernels ↑ during early ripening stages and ↓ as the kernels matured.<br><br>UPLC-Q/TOF-MS: identified 40 phenolic compounds. First reports of ellagic acid rhamnoside & rutinoside in pecans<br><br>New phenolics in pecans<br>6 compounds (e.g., bis-HHDP-glucose, glansreginin A & B, methyl gallate) first reported<br><br>(+)-Catechin most abundant in immature kernels \| Likely converts to proanthocyanidins during maturation<br><br>Antioxidant ∝ correlation<br>with TPC, particularly due to catechins & ellagic acid derivatives |
| Robbins KS, 2014 [28] | Variety Phenolics Characterization | Fractionation of crude extracts<br><br>TPC | Eighteen pecan cultivars were acquired from | USA | Major phenolics<br>Ellagic acid & (+)-catechin dominant in nutmeat with variations in LMW & HMW fractions |

| | | | | | |
|---|---|---|---|---|---|
| | | HPLC-ESI-MS/MS | commercial and research pecan orchards in Georgia (11), New Mexico (2), and Texas (5) | | PAC profile<br>Monomers to hexamers identified; dimers most abundant<br><br>Chromatographic profiling<br>Sephadex LH-20 enabled detailed phenolic separation<br><br>LMW fraction<br>No phenolic acids in final elution; rich in PAC monomers & dimers<br><br>HMW fraction<br>Primarily PAC dimers; also contained mono- to hexamers |
| Flores-Córdova M, 2017 [30] | Phytochemical composition | TPC<br><br>AOX: DPPH<br><br>Identification and quantification of phenolic compounds by LC-MS | 2 varieties<br><br>Western<br>Wichita | Mexico | Phenolics & tannins (7–8× ↑)<br>in shells vs. edible kernel<br>(Phenolics: 170.36 mg/g, Tannins: 582.07 mg/g)<br><br>↑ Phytochemicals<br>Wichita > Western<br><br>↑ AOX<br>Strong correlation with phenolics ($r^2 = 0.99$)<br><br>Detected: Gallic acid, ellagic acid, catechin<br>Ellagic acid absent in Western shells<br><br>Pecans rich in:<br>Phenolics, condensed tannins, hydrolysable tannins (gallic & ellagic acid, catechin)<br><br>→ Pecan shells = natural antioxidant source<br>Potential use in food & pharma industries |

| | | | | | |
|---|---|---|---|---|---|
| Robbins KS, 2015 [31] | Phenolics | TPC<br><br>Total procyanidins content (TPrC)<br><br>AOX: ORAC and FRAP<br><br>Tocopherol (T) and tocotrienol (T3) analysis<br><br>HPLC-ESI-MS | Eighteen pecan cultivars were acquired from commercial and research pecan orchards in Georgia (11), New Mexico (2), and Texas (5) | USA | TPC<br>1.82–2.62 g ellagic acid eq/100 g<br><br>Procyanidins<br>420–655 mg/100 g<br><br>Antioxidant capacity<br>H-ORACFL: 13.5–25.5 mmol Trolox eq<br>FRAP: 14.0–20.7 mmol $Fe^{2+}$ eq/100 g<br><br>Key phenolics:<br>Gallic acid, ellagic acid, proanthocyanidins<br>(No new aglycones post-hydrolysis)<br><br>Cultivar impact<br>Wichita = highest phenolics<br>Location = no major effect<br><br>↑ γ-tocopherols<br>15.98–27.73 mg/100 g<br>(Rich in lipophilic antioxidants) |
| Jia X, 2023 [32] | Metabolomics<br>Storage<br>Cultivar | TPC<br><br>AOX: DPPH and ABTS<br><br>UPLC-LTQ-Orbitrap $MS^n$ analysis<br><br>4 types of Storage conditions | Mahan | China | Phenolic diversity<br>118 compounds; 9 newly identified in pecan<br><br>TPC & AOX:↓ over storage time<br><br>↑ HT changes (fresh kernels)<br>↑ CT changes (dry kernels)<br><br>↔ Key metabolite markers (0d vs. 90/180d):<br>Catechin, PA trimer/tetramer, trigalloyl |

| | | | | | |
|---|---|---|---|---|---|
| | | Storage time: 0, 90 and 180 days<br><br>Storage temperature: 0°C and room temperature at 60 % RH | | | HHDP glucose, tetragalloyl hexoside → Indicators of quality deterioration |
| Rábago-Panduro LM, 2020 [33] | Bioactive compounds Storage | Storage experiment (4°C and 25°C)<br><br>Moisture content<br><br>Water activity (aw)<br><br>Lipid content<br><br>Fatty acids profile (GC-FID)<br><br>Tocopherols content (HPLC-PDA)<br><br>TPC<br><br>TF<br><br>CT<br><br>AOX: DPPH and ORAC | Western variety<br><br>In-shell wet pecans<br><br>In-shell dry pecans | Mexico | Drying process ↓ TPC (–33.2%) & antioxidant capacity (–22.3%)<br><br>↓ Condensed tannins –31.5% (kernels), –41.8% (in-shell) during storage<br><br>↑ DPPH antioxidant capacity +216.4% (kernels), +188.4% (in-shell) during storage<br><br>↑ γ-tocopherol retention Dry kernels: 73.1% vs. Wet kernels: 52.8% (after 210 days)<br><br>↑ Phenolic preservation In-shell pecans at 4°C (due to polyphenol migration)<br><br>↔ Tannin degradation correlated with ↑ DPPH Suggests polymerization boosts antioxidant activity. |

| | | | | | |
|---|---|---|---|---|---|
| Pham T, 2023 [35] | Storage<br>Cultivar | Color<br><br>TCC<br><br>TPC<br><br>HPLC-UV<br><br>Total Lipid<br><br>Texture | 5 cultivars<br><br>an improved (Pawnee), a native selection (Prilop of Lavaca or Prilop), and three breeding lines (1992-09-0041, 1992-090045, 2000-01-0027) | USA | ↑ Redness, ↓ yellowness<br>Color darkened during storage<br><br>↑ Lightness (dorsal side)<br>compared to ventral side of kernel<br><br>↓ Total carotenoids<br>↔ No significant change in total phenolics<br><br>↑ Gallic acid, catechin, ellagic acid<br>after storage<br><br>↓ Moisture content<br>↔ No change in total lipids<br><br>↔ Texture parameters (5)<br>Irregular changes in hardness, toughness, slope, fracturability, break<br><br>Genotypic variation observed (5 varieties)<br>Storage was the dominant influencing factor |
| Tan W, 2025 [34] | Food Quality<br>Storage | Moisture variation in fresh pecans<br><br>Mechanical shelling efficiency<br><br>Analysis of fat, protein, antioxidant content before/after shelling<br><br>Packaging methods tested for quality | Fresh pecan nuts (cultivar "Wichita") | China | Moisture content<br>Higher moisture → Improved mechanical shelling efficiency, reduced kernel breakage<br><br>Nutritional impact<br>Minimal effect on fat & protein levels<br><br>Antioxidant content<br>Slight ↓ in higher moisture pecans after shelling<br><br>Packaging methods<br>Vacuum packaging → Most effective for maintaining shelled pecan quality & shelf life |

| | | | | | |
|---|---|---|---|---|---|
| | | and shelf life | | | Optimal moisture content → Essential for efficient shelling \| Packaging → Key for quality preservation" |
| Bouali I, 2020 [36] | Phenolics<br>AOX<br>Cultivar<br>Harvest year<br>Carbohydrates | TPC<br><br>TFC<br><br>Total flavanols<br><br>TCT<br><br>Total o-diphenols<br><br>Phenolic compounds - HPLC-DAD<br><br>AOX: DPPH and FRAP<br><br>Carbohydrates: HPLC-RI | 3 cultivars<br><br>Mahan<br>Moore<br>Burkett | Tunisia | Mahan cultivar<br>↑ phenolics, flavonoids, flavanols, tannins, sugars & antioxidant activity<br><br>Major phenolics<br>Gallic acid, catechin, epicatechin, ellagic acid & derivative<br><br>Condensed tannins<br>Most abundant phenolic class<br>Ellagic acid<br>Most abundant compound<br><br>Antioxidant activity<br>Correlated with phenolic content<br><br>Composition variation<br>By cultivar & harvest year → impacts nutritional value |
| Noperi-Mosqueda LC, 2019 [37] | Fertilization<br>Quality | Fertilization treatments included different doses of nitrogen, phosphorus, potassium, calcium, liquid humus, and solid humus, applied using a Taguchi L25 | Schley | Mexico | Fertilization strategy<br>Mineral + organic fertilization \| Improved pecan production & quality<br><br>Phenolic content & AOX<br>↑ with combined fertilization \| Potential health benefits<br><br>Influential nutrients<br>Nitrogen \| Impacted yield, nut size & |

| | | | | | |
|---|---|---|---|---|---|
| | | experimental design.<br><br>TPC<br><br>AOX- DPPH | | | quality<br>Potassium \| No significant effect<br><br>Optimal dose<br>Max yield (3.2 t/ha) \| 184 kg/ha nitrogen, 107.4 kg/ha phosphorus, 50 kg/ha potassium, 2777 L/ha liquid humus |
| Gong Y, 2020 [38] | Phenolics<br>Tree pruning | TPC<br><br>ORAC<br><br>HPLC | Western<br><br>Pecans-hand harvested over 3 consecutive years from 2012-2014 from a commercial orchard in New Mexico | USA | Pruned samples had high TPC and ORAC compared to unpruned<br><br>TPC and ORAC: High canopy height samples >middle>low<br><br>Pruned trees → Higher ellagic acid & derivatives concentrations<br><br>Mechanical pruning + higher tree canopy → Increased nut antioxidant properties |
| Lee JH, 2021 [39] | Nuts/seeds<br>Comparison | Proximate Composition and Minerals<br><br>Minerals-ICP-OES<br><br>TPC<br><br>TFC<br><br>AOX -DPPH & ABTS | 10 types nuts/seeds<br><br>Cashews<br>Macadamia<br>Peanuts<br>Almonds<br>Pistachios<br>Walnuts<br>Pecans<br>Brazil nuts<br>Pine nuts | Korea | TFC → Pecans > walnuts<br><br>AOX (ABTS assay) → Pistachios > pecans<br><br>Main Minerals → Ca, Mg, Fe, Zn, Cu (common in most seeds and nuts) |

| | | | | | |
|---|---|---|---|---|---|
| | | | Sunflower seeds | | |
| Abe-Matsumoto L, 2010 [40] | Phenolics<br>Nut comparison | Moisture content<br><br>Lipid content<br><br>TPC<br><br>AOX: DPPH<br><br>Ellagic acid-HPLC-DAD | 11 different kinds of fresh nuts including pecans | Brazil | Wide range in phenols & AOX capacity across 11 nut types<br><br>Walnuts & pecans = rich in ellagic acid<br><br>↑ AOX linked to ↑ phenols and/or ellagic acid |
| Frezza C, 2025 [42] | Metabolic profiling | NMR | 3 cultivars<br><br>Wichita, Stuart, Sioux | Italy | Metabolite profiling 31 metabolites identified\| 28 quantified<br><br>Species differentiation<br>Multivariate PCA → Clear separation of 3 species<br><br>Key metabolites<br>Scutellarein & GABA levels → Linked to climatic adaptation of original growing regions |
| Xu M, 2020 [43] | Metabolic profiling | Phenolic metabolites: UHPLC-LTQ-Orbitrap MS for qualification and UHPLC-QQQ-MS for quantification<br><br>AOX: DPPH, | Pawnee<br><br>Kernel (without testa), testa, shell, shuck, leaf, branch, bark, and flower were collected | China | Phenolic Metabolites (n=97) → Identified from 8 pecan organs:<br><br>57 hydrolysable tannins (dominant)<br>19 condensed tannins<br>1 complex tannin<br>18 flavonols<br>2 other compounds<br><br>Testa contains highest amount of tannins |

| | | | | | | |
|---|---|---|---|---|---|---|
| | | ABTS | | | | Significant variation in metabolite numbers across organs<br><br>Hydrolysable tannin was the dominant kind of phenolic metabolites in pecan. |
| Kellett ME, 2019 [44] | Cellular AOX comparison<br><br>Raw and roasted | Quantitation of the CAA assay<br><br>Cell cytotoxicity assay<br><br>AOX: ORAC and FRAP<br><br>TPC<br><br>Characterization and quantitation of phenolic components (HPLC-ESI-MS/MS) | Desirable<br><br>Raw in shell | USA | | Phenolics ↓ Fluorescence (CAA assay)<br>Raw: –37–69%, Roasted: –26–68%<br><br>Active phenolics<br>Epicatechin dimers & trimers (560–840 g/mol) → likely cellular uptake<br><br>AOX: Roasting had no significant effect<br><br>CAA activity: High-molecular-weight (HMW) > Crude > Low-molecular-weight (LMW)<br><br>↔ No cytotoxicity<br>Pecan phenolics safe in Caco-2 cells |

| | | | | | |
|---|---|---|---|---|---|
| Gong Y, 2017 [45] | Phenolics<br>Extraction<br>Purification | Crude extracts were purified using Sephadex LH-20 column chromatography to enrich ellagitannin fractions.<br><br>Identification and characterization<br>HPLC-DAD-MS | Desirable<br><br>Chinese Hickory nuts | USA | Ellagitannins separation<br>Fused-core HPLC | Used in U.S. pecans → Chinese hickory nuts<br><br>Main ellagitannins<br>Tellimagrandin I, pedunculagin I & II | Found in both nut types<br><br>Ellagitannin concentration<br>Chinese hickory nuts > U.S. pecans<br><br>All ellagitannins → Strong AOX |

Arrows: ↓ (decrease), ↑ (increase), ↔ (no effect)

AOX: Antioxidant activity, ABTS: 2,2'-azino-bis(3-ethylbenzothiazoline-6-sulfonic acid), CAA: Cellular Antioxidant Activity assay, CT: Condensed tannins, DPPH: 2,2-diphenyl-1-picrylhydrazyl radical scavenging assay, FRAP: Ferric reducing antioxidant power assay, HPLC: High-performance liquid chromatography, HPLC-DAD: High-performance liquid chromatography with diode-array detection, HPLC-ESI-MS: High-performance liquid chromatography with electrospray ionization mass spectrometry, ICP-OES: Inductively coupled plasma optical emission spectrometry, LC-MS: Liquid chromatography–mass spectrometry, NMR: Nuclear magnetic resonance spectroscopy, ORAC: Oxygen radical absorbance capacity assay, PACs: Proanthocyanidins, PDA: Photo-diode array, TCT: Total condensed tannins, TF: Total flavonoids, TFC: Total flavonoid content, TPC: Total phenolic content, UPLC: Ultra-performance liquid chromatography, UHPLC: Ultra-high performance liquid chromatography
